# Human cumulative culture and the exploitation of natural phenomena

**DOI:** 10.1098/rstb.2020.0311

**Published:** 2022-01-31

**Authors:** Maxime Derex

**Affiliations:** CNRS, Institute for Advanced Study in Toulouse, University of Toulouse 1 Capitole, France

**Keywords:** cumulative culture, technology, innovation, social learning, cultural evolution, natural phenomena

## Abstract

Cumulative cultural evolution (CCE)—defined as the process by which beneficial modifications are culturally transmitted and progressively accumulated over time—has long been argued to underlie the unparalleled diversity and complexity of human culture. In this paper, I argue that not just any kind of cultural accumulation will give rise to human-like culture. Rather, I suggest that human CCE depends on the gradual exploitation of natural phenomena, which are features of our environment that, through the laws of physics, chemistry or biology, generate reliable effects which can be exploited for a purpose. I argue that CCE comprises two distinct processes: optimizing cultural traits that exploit a given set of natural phenomena (Type I CCE) and expanding the set of natural phenomena we exploit (Type II CCE). I argue that the most critical features of human CCE, including its open-ended dynamic, stems from Type II CCE. Throughout the paper, I contrast the two processes and discuss their respective socio-cognitive requirements.

This article is part of a discussion meeting issue ‘The emergence of collective knowledge and cumulative culture in animals, humans and machines’.

## 1. Introduction

What is the difference between a spear-thrower and a bow? Both are long-range weapons that allow hunters to throw projectiles at high velocity. Yet the underlying principles at work, i.e. the *natural phenomena* they exploit, clearly differ. The throwing power of both technologies stems from using leverage to amplify an input force and produce a greater output force. Bows, however, additionally exploit the elastic properties of materials such as wood to store elastic energy and convert it to kinetic energy.

Natural phenomena are not created by individuals; they are simply features of our environment. Yet they generate reliable effects, resulting from the laws of physics, chemistry or biology, that can be exploited and used for a purpose. As argued by the economist Brian Arthur, ‘[natural] phenomena are the indispensable source from which all technologies arise. All technologies, no matter how simple or sophisticated, are dressed-up versions of the use of some effect—or more usually, of several effects’ [1, p. 47]. Simple technologies such as cutting stone tools, for instance, exploit the effect of hard and sharp edges to slice through softer materials. Other technologies such as wheels harness the effect of rolling to facilitate movement by reducing friction.

There is nothing particularly remarkable about the fact that human cultural evolution exploits natural phenomena. Products of biological evolution also exploit natural phenomena. The teeth of carnivores are hard and sharp to cut through flesh, the structure of down feathers traps air to limit heat loss from birds’ body, and so on. Moreover, non-human animals also exploit natural phenomena through culturally acquired behaviours. Chimpanzees, for instance, socially learn to exploit the crushing power of heavy stones to crack nuts open [2].

Yet our ability to both expand the range of interdependent natural phenomena that we exploit and pass on the means of exploiting these phenomena to others is ultimately what underlies the open-ended dynamic that characterizes human cumulative culture. Indeed, any cultural trait that exploits a fixed set of natural phenomena can only be improved up to a point where it will run into some limitation. An individual equipped with a cutting tool can improve the perforating power of a wooden hunting spear by making it sharper. However, for the tool to be functional, a compromise will have to be made between sharpness and robustness. This will result in an optimal shape whose perforating power will be limited by the properties of the raw material. Once this fixed optimum is reached, there will be no more room for improvement, except to exploit additional natural phenomena to circumvent those limitations. Heating treatment, for instance, can be used to reach a new optimum by altering the structure and properties of the raw material [3].

Improving cultural traits that exploit a given set of natural phenomena (which is more akin to an *optimization* process [4,5]) and recruiting additional natural phenomena to push their improvement further and/or create new cultural traits (which is more akin to an *innovation* process) are two radically distinct processes, which, I shall argue, constitute the dual drivers of human cumulative cultural evolution (CCE). Indeed, both processes can generate the type of gradual improvement that characterizes cumulative culture. Yet optimization alone (which I will call Type I CCE) cannot lead to the emergence of new cultural traits and has only limited scope for pushing forward their efficiency. In comparison, the exploitation of an increasing number of natural phenomena (which I will call Type II CCE) bears the possibility of generating myriad cultural traits and continuous improvement.

The idea that human cumulative culture operates by exploiting an increasing range of natural phenomena is implicit in many arguments about the role of CCE in the emergence of increasingly complex cultural traits and more generally in arguments about the relationship between CCE and the ecological success of the human species [6–8]. Yet discussions about the socio-cognitive requirements of CCE have widely occurred as if it were a unitary phenomenon, which has led to much misunderstanding within the field of cultural evolution [9].

In this paper, I highlight the overlooked role of the cultural exploitation of natural phenomena in human CCE. I begin by contrasting Type I from Type II CCE and show why exploiting an increasing number of natural phenomena is a requirement for the continuous improvement of cultural traits. I outline the role of exploiting natural phenomena in the emergence of most features of human CCE, including cultural diversity and cultural dependency. I then highlight the role of exploiting natural phenomena in cultural domains such as art and belief systems. I discuss the collective processes that support the exploitation of an increasing number of natural phenomena by human populations. Finally, I discuss the socio-cognitive requirements of Type I and Type II CCE.

## 2. Distinguishing between Type I and Type II cumulative cultural evolution

In this section, I present a few documented examples of cultural optimization in both non-human and human animals and highlight how Type I and Type II CCE both participate in the gradual improvement of cultural traits.

### (a) Type I cumulative cultural evolution

A well-known example of Type I CCE in non-human animals comes from the experimental work of Sasaki & Biro [10]. In their experiment, pairs of homing pigeons had to solve a navigation task across successive artificial ‘generations’, with the most experienced bird of a pair replaced by a naive bird at each generation. Their results show that pairs of pigeons that undergo the sequential replacement of their members develop increasingly efficient flight routes over time and outperform fixed-pairs of pigeons. Such an example fulfils all the four criteria that Mesoudi & Thornton have highlighted in their recent review discussing the notion of CCE: (i) change in behaviour, (ii) transfer via social learning, (iii) improvement in performance, and (iv) sequential repetition of the first three criteria [9]. Yet a notable feature of this specific instance of CCE is that improvement cannot keep going indefinitely. Indeed, the navigation task that pigeons have to solve is associated with an optimal, fixed solution (i.e. the bee line path), which, once found, leaves no more opportunity for improvement [10].

This example does not involve harnessing natural phenomena other than those that biological evolution has allowed pigeons to exploit (such as lift). Yet finite optimization will also occur with tasks that involve the cultural exploitation of a limited number of natural phenomena. For instance, in a recent experiment where human participants were asked to optimize a wheel that had four radial spokes and one weight that could be moved along each spoke, two natural phenomena could be manipulated by participants (i.e. the effect that objects whose mass is distributed closer to their axis of rotation require less torque to increase angular momentum and the effect that initial torque can be increased by unevenly distributing mass) [5]. Results of this experiment show that, across successive artificial generations of participants, wheels get progressively faster at covering a given distance. Yet, because the collective search process takes place within a finite space, there also exists a solution, which, once found, leaves no more room for improvement.

Most tasks that have been used to study CCE among human participants share this feature of finite optimization (e.g. [11–16]). In experiments where participants make paper airplanes so that they fly as far as possible, the collective search takes place within a fixed space that consists of all the shapes that can be produced from a single sheet of paper [11]. It is worth noting that tasks where people can produce an infinity of solutions have sometimes been considered as ‘open-ended’. Reindl *et al.*, for instance, presented a task where children had to build something as tall as possible using a given amount of plasticine and a limited number of sticks as open-ended [17]. Yet, as demonstrated by the homing pigeons' navigation task, the possibility of generating an infinity of solutions does not mean that continuous improvement can occur. Providing individuals with limited material makes the search space inherently finite, creating a fixed optimum and preventing the possibility of continuous improvement. Importantly, making the search space larger is not a sufficient condition for creating additional opportunities for improvement. For instance, providing participants with grains of rice in addition to plasticine and sticks will theoretically expand the search space in Reindl *et al*.'s experiment. However, this will not allow participants to produce taller solutions. This is because using grains of rice will not permit individuals to harness additional natural phenomena relevant to the task at hand. If this were the case, a new period of optimization towards a novel optimum would follow. As we shall see below, only when CCE exploits an increasing number of natural phenomena does it lead to an open-ended cultural dynamic and continuous improvement.

### (b) The dual drivers of continuous improvement

Let us take the example of a spear to illustrate why exploiting an increasing number of natural phenomena is a requirement of continuous improvement and open-ended CCE. Observational studies have shown that chimpanzees bit the tip of branches to produce crude spears to hunt bushbabies [18]. Let us assume, just for the sake of argument, that chimpanzees would greatly benefit from making spears sharper. How could that be achieved? Crude biting procedures would probably emerge first, initially leaving much room for improvement through Type I CCE (i.e. cultural optimization). After some time, refined, multi-step biting procedures might emerge, pushing the efficiency of spears forward. Assuming human-like social learning abilities, steps from different biting procedures might be combined by individuals attending to multiple cultural demonstrators and promote the emergence of even sharper spears. Yet, after some time, opportunities for improvement will start to dry up as individuals will move closer to a fixed optimum. Indeed, biting branches eventually causes fibres to become crushed and damp, which ultimately sets an upper limit to the perforating power of spears produced in this manner. No amount of biting, expertise in biting, faithfulness in biting skills transmission, or combination of biting methods would allow chimpanzees to get beyond this upper limit.

What chimpanzees would need to further increase the perforating power of spears is to harness additional natural phenomena, such as exploiting the cutting power of sharp edges to slice through fibres. Using sharp-edged flakes would allow fibres to remain dry and stiff and permit individuals to produce spears with previously unattainable perforating capabilities. The removal of initial constraints will pave the way for a new period of cultural optimization by which sharpening procedures will be improved. After a while, opportunities for improvement will start to dry up again as solutions approach the new fixed optimum. The tip of very sharp spears, for instance, will be prone to failure, which will force individuals to balance perforating capabilities with robustness. At this point, new natural phenomena must be harnessed to push the efficiency of spears further (such as the hardening power of heating treatments [3]).

The evolutionary history of any single technology, no matter whether it is simple or complex, is consistent with the view that additional natural phenomena must be harnessed to continuously improve the use of a *base natural phenomenon*. Take a more recent example such as the bicycle. Like its modern counterpart, the ancestor of the bicycle had two wheels and thus exploited the base natural phenomenon that rolling facilitates motion by reducing friction. Early solutions called draisiennes were powered by the rider's feet on the ground and could not achieve great speeds. Improvement through Type II CCE came in the form of pedals and cranks attached directly to the front wheel, which allowed cyclists to use leverage to set the bicycle in motion. The exploitation of this additional natural phenomenon paved the way for a new period of Type I CCE, whereby bicycles were progressively equipped with unreasonably large front wheels (which allowed riders to cover a greater distance per crank revolution) [19]. This solution, however, made bicycles dangerously unstable, which forced bicycle manufacturers to balance stability and speed. Eventually, a way was found to decouple the size of the wheel from the speed of the bicycle by introducing the chain drive and driven sprockets into its design (which is an improvement that uses multiple natural phenomena and results from Type II CCE).

**Figure 1.**
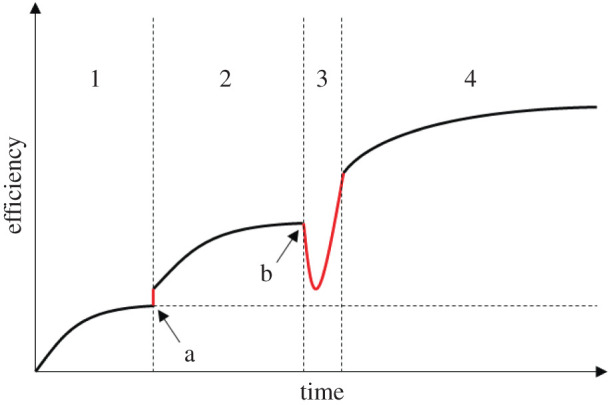
Illustration of how Type I and Type II CCE may affect the improvement of a single cultural trait. In the first period (1), the solution undergoes Type I CCE (i.e. cultural optimization). Opportunities for improvement progressively dry up as the solution approaches a fixed optimum. The horizontal dotted line illustrates the maximum efficiency that can be reached when Type I CCE is the only process at play. The exploitation of an additional natural phenomenon (i.e. Type II CCE) by chance (a) boosts the efficiency of the trait beyond the previous optimum at no cost to the innovator and expands the search space. This triggers a new (and typically longer) period of cultural optimization (2). Integrating an additional natural phenomenon functionally (3) incurs costs to the innovator (b) before eventually boosting the efficiency of the trait and paving the way for a new period of cultural optimization (4). The socio-cognitive capacities underlying (a) and (b) are discussed in §6. (Online version in colour).

Thus, Type I and Type II CCE have different implications for the evolutionary dynamics of cultural traits. Type I CCE is a process by which solutions get gradually closer to a fixed optimum but cannot get past this point. Type II CCE is a process by which existing constraints are removed through the harnessing of additional natural phenomena. It results in a modified, expanded search space and paves the way for new periods of cultural optimization. Ultimately, the possibilities for continuous improvement and open-ended CCE stem from the harnessing of an increasing number of natural phenomena (figure 1).

## 3. Type II cumulative cultural evolution can generate endless cultural traits

I have argued that the possibility of generating continuous improvement requires harnessing an increasing number of natural phenomena, but human culture is also characterized by an increase in the diversity of cultural traits over time [20–23]. Because Type I CCE consists in optimizing an existing cultural trait within a finite search space, it is unlikely to lead to long-lasting cultural diversity. Founder effects might result in different evolutionary trajectories, but eventually different populations should converge toward the same solution. Convergence between different experimental chains or groups of participants has been observed in tasks involving the cultural optimization of paper airplanes, spaghetti towers and virtual fishing nets [11,24], as well as between participants taking part in collaborative programming competitions [25].

By contrast, Type II CCE will drive up cultural diversity in the long run. One obvious reason is that there exist different natural phenomena that afford different functionalities. Exploiting the cutting power of hard and sharp edges on softer materials, for instance, requires a different solution than the one required to exploit the crushing power of heavy materials on softer materials. Another reason is that different solutions to the same problem can recruit different natural phenomena. For instance, there exist two basic methods to produce fire. One consists in using the friction of hard wood on soft wood to produce a hot coal; the other consists of striking flint against pyrite to produce sparks [26]. The most important reason, however, is that exploiting natural phenomena facilitates the discovery of previously unavailable natural phenomena, the use of which can be incorporated into cultural repertoires, combined with other natural phenomena, or exapted to new purposes.

### (a) Uncovering natural phenomena

The main way by which Type II CCE can promote the harnessing of additional natural phenomena is by changing individuals' direct environments. Take chimpanzees cracking nuts or capuchin monkeys breaking stones to ingest powdered quartz or lichens [2,27]. Both behaviours exploit the crushing power of hard and heavy material and have been shown to lead to the unintentional production of sharp-edged flakes (similar to those observed in early hominin technological repertoires). This indicates that harnessing the crushing power of hard and heavy material can make individuals more likely to discover the cutting power of sharp edges by making appropriate material more available in individuals’ direct environment.

The use of fire provides another example of how the exploitation of natural phenomena can help to uncover additional natural phenomena. Initially, naturally occurring fires could have created foraging opportunities for early hominins by removing obstacles to locomotion and prey detection and creating patches where food items (such as insects and shallow-rooting tubers) could more easily be located and collected [28]. Early hominins may have taken advantage of this natural phenomenon and transported fire from burnt to unburnt areas. This, in turn, would have increased the amount of time hominins spent near fire, making them more likely to witness natural phenomena that result from the effect of fire on diverse materials: unintentionally burnt items might have revealed the detoxifying and/or predigesting power of cooking [29], the hardening power of fire on fibrous material [3], the adhesive power of the by-products of burnt fibrous material [30] and the enhanced flake-ability of heated stones [31]. The regular exploitation of natural phenomena for a given purpose, such as landscape manipulation, can reveal many other natural phenomena.

### (b) Combining and exapting natural phenomena

Another way by which the exploitation of natural phenomena can promote cultural diversity is by creating combinatorial opportunities. Combination has often been put forward as a mechanism promoting CCE [32–36]. Yet, this notion has been used inconsistently in the literature. For instance, Kempe & Mesoudi [12] showed that participants completing jigsaw puzzles can combine information when exposed to the solutions of different cultural demonstrators. However, what Kempe and Mesoudi documented is more akin to an *aggregation* phenomenon whereby individuals pool together partially completed solutions to the same problem (see also [15]). Furthermore, aggregation has limited influence on cultural evolutionary dynamics as it does not modify the fixed optimum towards which a population is expected to evolve through Type I CCE. In other cases, the notion of combination has been applied to the different elements of cultural repertoires [21,36–38]. Yet, as pointed out by Arthur, novel technologies are not literally made of pre-existing technologies [1]. Hunting bows might have evolved because bow drills and spears were both part of the cultural environment of individuals. However, hunting bows are not the product of the combination of a bow drill and a spear. What must be combined for the solution to be useful are the functionalities afforded by the natural phenomena exploited and/or uncovered by those pre-existing technologies. This is important since it means that combining existing principles will usually require individuals to pay costs in terms of time and energy to modify existing traits and integrate them functionally (see §6 for implications for the cognitive requirements to Type II CCE). Even though theoretical models have abstracted away the details of how combination takes place effectively, results remain valid regarding the role that combination plays for CCE. In particular, it has been shown that combination leads to a phenomenon known as combinatorial explosion [39,40] and can promote an exponential increase in cultural repertoire size [37].

Importantly, the combination of natural phenomena not only occurs between previously exploited natural phenomena, or between previously exploited and recently uncovered natural phenomena. It can also occur between previously exploited and previously known but useless natural phenomena. Indeed, many natural phenomena are of no immediate adaptive value in themselves. Take the adhesive power of some substances or the energy storage capabilities of elastic materials. Both are natural phenomena that are readily available in nature but useless when used in isolation. Yet, once other natural phenomena have been harnessed and incorporated into the cultural repertoire of a population, previously useless natural phenomena become a potential source of innovation. For instance, once wooden spears and stone tools are both part of the same cultural repertoire, the adhesive power of some substances can be harnessed to strongly hold stone points in place and push the efficiency of spears forward [41,42]. Sometimes, many natural phenomena must be harnessed before the use of a known natural phenomenon can be incorporated into the cultural repertoire of a population. For instance, ancient Greeks discovered static electricity by witnessing that amber attracts light materials when rubbed [43] (records also show that ancient Egyptians were familiar with electric fishes [44]). Yet because not much can be obtained from this natural phenomenon before harnessing many others, electricity would remain no more than an intellectual curiosity until 1600.

Still another way by which the effects of natural phenomena can promote CCE is by being repurposed to a new end. Muthukrishna & Henrich [33], for instance, have pointed out that the principle of storing elastic energy led to the invention of bows, spring traps and string instruments. Interestingly, the authors have noticed that those three cultural traits were never developed in Australia where the principle of stored elastic energy was never discovered.

These examples illustrate how harnessing natural phenomena opens new ‘thought spaces’ [33] and gives rise to an ever-expanding space of possibilities, with previously inaccessible solutions suddenly becoming part of a new ‘adjacent possible’ [45]. Importantly*,* because natural phenomena are not scattered randomly but form a network of effects with uneven numbers of connections (harnessing fire, for example, can reveal many more natural phenomena than harnessing probing sticks), Type II CCE can result in punctuated rather than gradual cultural accumulation. This is illustrated by recent theoretical models showing that puzzling cultural evolutionary patterns such as periods of stasis interspersed by sudden increases in cultural traits need not depend on exogenous factors such as abrupt changes in population size and connectedness [46,47]. Instead, sudden increases in cultural complexity can result from the emergence of cultural traits that facilitate the emergence of other related traits [21,37,48].

### (c) Type II cumulative cultural evolution generates cultural dependencies

The fact that natural phenomena are not readily available but must be progressively uncovered using solutions that harness less deeply hidden natural phenomena creates cultural dependencies, which some have argued is the core of what CCE is about [6,21,37,49,50]. This contrasts with solutions accessible through Type I CCE that need not depend on pre-existing solutions. Take the wheel experiment mentioned above for instance [5]. An individual with flat priors about the task will have the same probability of producing the least good and best solutions since all configurations have exactly 1 chance out of 20 736 to be produced (20 736 being the number of possible configurations). Because the probability of producing the best solution is small, multiple trials will usually be necessary for individuals to reach this optimum. More generally, when the search space is larger than what individuals can search individually, gradual improvements should occur over successive generations of individuals linked by cultural transmission (as illustrated by the homing pigeons and wheel experiments). Yet, whether it is the beeline in the homing pigeons' navigation task or the best configuration of weights in the wheel experiment, optimal solutions fundamentally remain within the reach of naïve individuals. Some might argue that the same is true of Type II CCE, since there exists a sequence of actions that individuals might perform by chance and will result in the production of a bow. Yet, as pointed by Erwin [51], this perspective assumes that the search space pre-exists rather than being constructed over time. From a behavioural perspective, and contrary to what is often assumed, the probability of building a bow by chance is not close to 0 but effectively 0. This is because there is no incentive for entirely naïve individuals to perform the energetically costly sequence of actions that is required to produce a bow. By contrast, there exist incentives for naïve homing pigeons for going from point A to point B, and so an associated probability (however small) for them to navigate along the shortest possible path by chance. Incidentally, this means that solutions produced through Type I CCE cannot theoretically be beyond what a single individual could have produced alone, which is sometimes considered as a criterion for CCE [52].

## 4. Natural phenomena across cultural domains

In the previous section, I argued that harnessing natural phenomena paves the way for the open-ended dynamic that characterizes human cumulative culture. However, so far, I mostly focused on material artefacts. What about CCE in other domains such as art and belief systems? Do immaterial cultural traits result from the same two processes of Type I and Type II CCE?

As argued by Arthur, immaterial cultural traits tend to be perceived differently than material cultural traits because they are based upon different types of natural phenomena [1]. Yet they too exploit natural phenomena. The functioning of our brain, for instance, involves many natural phenomena that can be taken advantage of through Type II CCE. Animation studio's artists, for instance, tweak light and colour to trigger deep emotional responses [53]. Social media platforms exploit our need for social interaction and introduce features that stimulate our brain's reward network [54]. Groups that oppose genetically modified organisms (GMO) develop arguments that appeal to our intuitive expectations to raise and amplify concerns about GMO use [55]. Magicians exploit perceptual and cognitive principles governing how we reason about things to fool us and create magical experiences [56]. Still other types of natural phenomena are exploited in domains such as medicine and culinary practices, namely the natural phenomena of our bodies. Thus, even though the natural phenomena of our brain and body are not as fixed as physical laws and are subject to gene-culture coevolution, they still generate reliable effects that can be exploited and used for a purpose.

One implication of this is that natural phenomena like those resulting from the specific architecture of our brains can be progressively uncovered and exploited by Type II CCE to produce increasingly appealing cultural traits. This is illustrated by studies that have revealed consistent patterns behind cultural products, behaviours or beliefs that, at first glance, seem dissimilar [57–60]. For instance, rituals that are used to treat problems are very diverse in the sense that they involve the use of different substances, artefacts and practices. Yet, they are also very similar in the sense that they often exhibit features such as procedural repetition (e.g. action *x* must be repeated three times), a large number of procedural steps (e.g. do *x* then *y* then *z*) and time specificity (e.g. *x*, *y* and *z* must be performed during full moon) [57]. In a study conducted among Brazilian and United States participants, Legare & Souza [57] experimentally manipulated the content of rituals to evaluate how common features of rituals affect participants' evaluation of their efficacy. Their results show that rituals specifying time, involving longer repetition of procedure and entailing greater number of steps are rated as more efficacious than rituals that do not specify time, involve fewer repetition of procedure and entail fewer number of steps, respectively. Based on these results, Legare and Souza have argued that common features of rituals reflect our intuitive beliefs about causal relationship and the efficacy of goal-directed action sequences. In the framework of this paper, this suggests that immaterial cultural traits such as rituals can evolve in a way that allows them to tap into an increasing number of intuitive causal principles, which are natural phenomena of our brains. Modifications that exploit additional natural phenomena of our brains (such as the introduction of a repetition procedure into the content of a ritual) result from Type II CCE, while changes in the implementation of the use of a given natural phenomenon result from cultural optimization (such as the number of times a given action must be repeated or the artefacts and substances it involves).

Another reason why Type II CCE is critical to the gradual improvement of immaterial cultural traits is because material products resulting from Type II CCE make us better at exploiting the natural phenomena of our brains and bodies. Artistic products of CCE, for instance, keep becoming more appealing to human minds because novel material solutions offer new opportunities to ‘hack’ human brains. Artists from animation studios, for instance, have long used colour and light to convey both narrative and emotion. Yet, improvements in material solutions keep offering new opportunities for animation artists. Wide gamut colour screens, for instance, allow artists to use expanded colour spaces made of ‘greener’ greens, ‘redder’ reds, and so on to elicit stronger emotional responses than what was possible before [53]. Thus, while it has been argued that art results from cultural evolutionary processes that fall outside of the technological domain [61], improvements in artistic products of CCE often rely on improvements in material domains. More generally, most immaterial products of CCE only exist because of Type II CCE in material domains. The cultural evolution of mathematical knowledge, for instance, depends on material products of CCE, such as notepads and computers, that have pushed forward our ability to reason about mathematical objects. Similarly, early symbolic systems, such as markers of status or cultural identity, relied on means of producing durable vehicles to convey information, which required material products of CCE (to extract, crush and mix pigments or carve material).

## 5. Harnessing natural phenomena collectively

The fact that Type II CCE operates by harnessing an increasingly intricate web of interdependent natural phenomena that span across domains makes it a process that is inherently collective. Population size and structure have been widely put forward as factors that critically impact individuals' ability to develop increasingly efficient cultural traits [36,38,46,47]. Theoretical and experimental studies, for instance, have shown that larger groups produce greater cultural variation and suffer less cultural loss, which promotes the gradual improvement of cultural traits [15,16,46,47]. Although experiments testing the effects of demography on CCE mostly involve Type I CCE, similar effects should be observed when Type II CCE is involved. With Type I CCE, more cultural variation will lead to a greater probability of producing solutions close to the fixed optimum and more learners will reduce the risk of losing these improved solutions. With Type II CCE, more cultural variation will result in a greater probability of exploiting novel natural phenomena and more learners will decrease the risk of losing their use.

Type II CCE, however, depends on collective processes that go beyond learning from others and buffering the risks of cultural loss. Indeed, exploiting an increasing number of natural phenomena requires mastering an increasing amount of accumulated knowledge and know-how over time. This contrasts with Type I CCE in which solutions closer to a fixed optimum are not necessarily harder to learn (as illustrated by the homing pigeon experiment [10]). Theoretical models have shown that mastering an increasing amount of accumulated knowledge can impose constraints on CCE [62]. This is because, over time, learners need more time to acquire what has been discovered before. This effect is illustrated by a computer-based experiment in which chains of participants had to develop complex artefacts composed of an increasing number of sub-items [63]. For instance, participants could use various materials, such as pieces of wood and rock, to produce axes, then use axes to cut trees, and then use logs to build totem poles. Unsurprisingly, the results show that individuals who inherit more accumulated knowledge spent more time recreating inherited items and have less time left to innovate further [63]. Theoretical models have shown that, when acquisition costs increase over time, CCE may come to a point where individuals spend all their time acquiring what previous generations have discovered before, which can stall the process of cultural accumulation [62]. This suggests that, for Type II CCE to continue, populations must collectively find ways around this limitation, such as by distributing accumulated knowledge among ever wider networks of individuals working together towards a common goal (see [64] for a discussion about the role of joint goals for human CCE).

The pivotal role of collaborative networks in Type II CCE is illustrated by the results of a recent study that investigated the evolution of Hollywood film production crews over 100 years [65]. In their paper, Tinits and Sobchuk analysed film crew complexity over time using metrics such as the number of individuals involved in the production of a film and the number of unique job titles used within each film. Their results show that the mean number of people involved in a film grew from about eight in the 1910s to about 604 in the 2000s and the mean number of unique jobs from about seven to about 283 over the same period. Similar results have been observed in studies looking at the production of scientific knowledge. For instance, Wuchty *et al*. have analysed millions of scientific papers and patents over five decades and showed that teams are increasingly more likely to produce high impact research than solo authors [66].

The relationship between Type II CCE and the size of collaborative networks suggests that material and immaterial cultural traits must coevolve for Type II CCE to continue. Indeed, immaterial products of CCE such as symbolic systems, cultural norms and legal systems are a requirement for the collaboration of increasingly larger networks of unrelated individuals working together [67,68].

## 6. Socio-cognitive requirements to Type I and Type II cumulative cultural evolution

Considerable debate exists over the underlying socio-cognitive requirements of CCE [7,69–74]. Conflicting arguments and empirical evidence have been put forward about the role of evolved social learning mechanisms and causal reasoning in CCE [7,70,71,75]. This is widely owing to a failure to distinguish between the two distinct processes that underlie the open-ended dynamic that characterizes human CCE. Indeed, Type II CCE is likely to depend on socio-cognitive requirements that are over and above those involved in Type I CCE.

### (a) Type I cumulative cultural evolution

There are good reasons to think that myopic search processes can give rise to Type I CCE, which suggests that cultural optimization can emerge from widespread learning abilities. Indeed, reward sensitive individuals can progressively get closer to a fixed optimum as a result of learning errors or mistakes. An example in humans comes from a study looking at the evolution of air-resonance power efficiency in the violin [76]. In a violin, air flows from the violin's air cavity to the exterior via sound holes whose shape affects resonance efficiency. Over centuries, sound-hole geometry of the violin's ancestors slowly evolved from circles to f-holes, which resulted in a twofold increase in air-resonance power efficiency. Over two additional centuries, f-hole length slowly increased by roughly 30% resulting in an additional power increase of roughly 60%. Although the effect of intentional local exploration around pre-existing solutions cannot be excluded, the results indicate that the rate of evolution is consistent with the gradual accumulation of modifications resulting from copying errors and the selective retention of instruments with higher air-resonance power. The same process is likely to underlie results from recent laboratory and field experiments showing that causal knowledge is not a requirement for the emergence of culturally optimized solutions (which is also illustrated by the homing pigeon experiment) [5,77].

There are also reasons to think that the cultural transmission of beneficial modifications resulting from Type I CCE can rely on widespread social learning abilities. One reason is that culturally optimized solutions do not necessarily become harder to learn [78], nor more causally opaque over time. This is well exemplified by the homing pigeon experiment in which the optimal solution is less convoluted, and thus less complex, than less efficient ones [10]. Even when the exploitation of natural phenomena is involved, cultural optimization does not necessarily lead to harder-to-learn solutions. In the wheel experiment, for instance, the most efficient configuration is not harder to learn than the less efficient ones because both configurations depend on the position of the same four weights [5]. As a consequence, it is not surprising that some experiments involving Type I CCE have shown that evolved social learning mechanisms are not a requirement for the gradual improvement of cultural traits [69,70,79].

Thus, although evolved reasoning and social learning abilities can promote Type I CCE rates [5,79], there are few reasons to think this process of cultural optimization should only be found in a handful of species with specific socio-cognitive abilities.

### (b) Type II cumulative cultural evolution

Distinguishing between Type I and Type II CCE does not make the differences between human and non-human animal culture any less puzzling. The culture of non-human animals is less diverse and complex than that of humans, yet animals also exploit natural phenomena: the crushing power of heavy stones to extract nut kernels [80], the perforating power of branches to kill prey [18], the cleansing power of water to wash the sand off edible resources [81], the probing power of sticks to collect insects [82], the water retention power of moss to collect water [83], the hooking power of curved twigs to extract worms [84], and so on.

What these behaviours seem to have in common, however, is that (i) they rely on natural phenomena whose effects can be observed from manipulating readily available materials, and (ii) they have an immediate effect on the provision of resources. In that regard, they rely on natural phenomena similar to those exploited by early hominins: rocks were used to pound bones open for marrow, flakes (that might initially have been by-products of other activities [27]) were used for flesh removal, and so on. The fact that the natural phenomena exploited by non-human animals can result from the manipulation of readily available material and provide immediate benefits suggests that simple associative learning mechanisms may account for their incorporation into individuals' behavioural repertoires. For instance, an individual might accidentally crack a nut open by playing around with rocks. This lucky accident will deliver a reward to the individual and act as a reinforcer that will make the behaviour more likely to be repeated in the future [85]. This indicates that harnessing some natural phenomena may result from widespread cognitive abilities.

Importantly, a myopic process can even lead to the emergence of complex behavioural sequences involving multiple phenomena through small associative steps. For instance, once nut cracking is incorporated into the behavioural repertoire of individuals, serendipitous events might make them learn that it is more efficient to crack nuts on hard rather than soft surfaces. This might lead individuals to use anvils in the form of surface roots or loose rocks. This process through which complex behavioural sequences are established through small associative learning steps is called chaining [86]. Opportunities to harness an increasing number of natural phenomena at no cost to the innovator, however, depend on the topology of the expanding search space. Indeed, for Type II CCE to continuously operate through a combination of serendipitous discovery and associative learning, there must exist endless paths along which fully formed, increasingly rewarding solutions can emerge by chance.

Yet most natural phenomena are unlikely to provide benefits at no cost to the innovator. The energy storage capabilities of elastic materials can be discovered by messing around with readily available material yet witnessing this effect will not provide individuals with any benefits before being functionally combined with usable principles through trial-and-error. This has several implications for the cognitive abilities that might underlie Type II CCE. First, it suggests that not immediately useful natural phenomena must first be recognized by individuals as usable effects before being incorporated into the behavioural repertoire of individuals. This is likely to require abilities such as *functional representation,* which allows individuals to conceive an object or an event in relation to a future end-state [87]. Such an ability may allow individuals to harness natural phenomena with no immediate benefits on the grounds that they represent some means to a desired end. Abilities such as causal or technical reasoning are also likely to be involved, as they will enable individuals to recognize that the exploitation of a given natural phenomenon can cause a desired effect [71]. Second, it suggests that Type II CCE requires individuals to be willing to pay costs for hypothetical future benefits. This is likely to depend on an ability to pursue long-term goals, which might depend on capacities such as mental time travelling [88,89].

The proper transmission of beneficial modifications resulting from Type II CCE is also likely to require specific socio-cognitive abilities. As mentioned earlier, Type II CCE generates an ever-larger amount of accumulated knowledge and know-how, which will result in increasing selection pressures for securely passing the means of exploiting natural phenomena to others. Moreover, cultural traits resulting from the exploitation of several natural phenomena should be associated with an opaque mode of production. This suggests that sophisticated social learning mechanisms are likely to be key to the build-up of Type II CCE (as it has been often argued about CCE [20,74]). Sophisticated social learning mechanisms, however, might not be a sufficient condition to the proper transmission of traits resulting from Type II CCE. Indeed, since Type II CCE depends on harnessing natural phenomena that are not directly associated with perceived benefits, social learners will have no incentives to learn how to exploit them. One capacity that might help individuals to get past this bottleneck is *teleological action interpretations*, which consists in interpreting the actions we observe in functional terms [90]. Indeed, such an ability can promote social learning of novel means actions and novel goals, which is a requirement to the harnessing of not immediately useful natural phenomena.

## 7. Conclusion

The notion of CCE—originally defined as the process by which beneficial modifications are culturally transmitted and progressively accumulated over time—was first developed to identify what is distinctive about human culture and cognition [91]. In this paper, I have argued that two distinct processes satisfy the original definition of CCE: improving cultural traits that exploit a given set of natural phenomena (Type I CCE) and exploiting an increasingly larger number of natural phenomena (Type II CCE). The unparalleled diversity and complexity of human culture stems, I have argued, from the second process.

In their review discussing CCE, Mesoudi & Thornton distinguished between what they call a ‘core’ set of criteria for CCE and an ‘extended’ set [9]. The core criteria are both necessary and sufficient for the gradual improvement of cultural traits. They are (i) change in behaviour, (ii) transfer via social learning, (iii) improvement in performance, and (iv) the sequential repetition of the first three criteria. The extended criteria are not essential for the gradual improvement of cultural traits but are often found in paradigmatic cases of human CCE cited in the literature. They include: (i) functional dependence [21,50], (ii) diversification [22,92], and (iii) recombination [32–34,36]. A key unresolved question Mesoudi & Thornton discuss is what underlies the distinction between their core and extended criteria? My account of Type I and Type II CCE offers one solution. Mesoudi & Thornton's core criteria can be met when Type I CCE is the only process at play, while their extended criteria will be met in species that can culturally exploit an increasing number of interdependent natural phenomena.

Acknowledging the role of Type I and II CCE will help clarify debates about what underlies humans' ability to develop increasingly complex cultural traits. Indeed, the failure to distinguish between these two distinct processes has led experimenters (including me) to focus almost exclusively on Type I CCE while hampering debate about the socio-cognitive requirements to CCE. The critical role of Type II CCE in the diversity and complexity of human culture calls for new experimental tasks allowing individuals to uncover and harness an increasingly large number of natural phenomena. There is much to be gained from developing experimental tasks that better capture the challenges of open-ended CCE and investigating the creative and collective processes that support it.

## Data accessibility

This article has no additional data.
